# Supplementary material for: Medical advocacy in the face of Australian immigration practices: A study of medical professionals defending the health rights of detained refugees and asylum seekers
Source: PLoS One. 2020 Aug 21;15(8):e0237776. doi: 10.1371/journal.pone.0237776 (PMC7442262; doi:10.1371/journal.pone.0237776)
Supplement: S1 Table — (DOCX) [file pone.0237776.s002.docx]

| *Personal ethics and perspectives on role obligations* | |  |
| --- | --- | --- |
| Quote 1 | ‘I can give you a poetry quote which probably sums it up. Charles Kingsley. He said,  “Do the work that’s nearest / Though its dull at whiles, / helping, when we meet them, / lame dogs over stiles” ^^[[1]](#footnote-1)^^  So, I think that kind of sums up the way I feel about things, that I'm probably not one to go out and actively look for lame dogs to assist. But if I find a lame dog, if I find someone in need, then I feel it is my personal and professional responsibility to assist that person. And so, if I hear something, then I think I have a medical obligation and a personal obligation to assist as much as I can.’ *P31* | |
| *Patient centred care* | |  |
| Quote 2 | ‘I am always guided by principles such as first do no harm, and you know, the patient's well-being is the primary concern.’ *P30* | |
| Quote 3 | ‘Our duty is to the patient… my duty is to the patient.’ *P14* | |
| Quote 4 | ‘certainly, it's pretty clear for myself… that if you have a patient, then you have a duty to advocate for what's best for that patient.’ *P01* | |
| Quote 5 | ‘when you do the medico legal report… your duty is to the court… you’re not to be an advocate. You're just to tell the court what's going on… So, I phoned the lawyers and said, “look, you know, I know that you said that, that I'm just to report to the court… But I actually have a duty of care in that I think this person's is at risk of death. And I've seen them now and so I kind of feel like I have to do something about it.” And they were like, “Oh, ok, give me a minute.” So, they phoned some legal ethics centre, and then they phoned me back. And they said, “Well, you gotta do what you have to do. And can you give us the report before you do anything else?”’ *P30* | |
| *Social determinants of health* | | |
| Quote 6 | ‘Do you feel like healthcare professionals have a responsibility to address social conditions that impede health?’ *Interviewer*  ‘Oh absolutely. I don't know how you can be a doctor and not address those things.’ *P06* | |
| Quote 7 | ‘…psychiatrists who don't work in that model are just not practicing good psychiatry, and a lot of them don't work in that model. They work in the 'here take this pill' model. And I look at the broader issues… we need to be thinking more broadly.’ *P09* | |
| Quote 8 | ‘[colleagues think that] doctors and the professional medical body should confine themselves to, for example, the quality of healthcare service providers, or the diagnosis of the individuals, rather than talking about the context so much.’ *P28* | |
| Quote 9 | ‘Do think that health care professionals have a responsibility to address social conditions that impede health? *Interviewer*  ‘I absolutely do. I don't think we are trained in that though. I think we are very much trained in acute medicine.’ *P14* | |
| Quote 10 | [Referring to the social determinants of health] ‘I don't think it was covered particularly well as a concept in the medical school curriculum.’ *P06* | |
| Quote 11 | ‘And I think that's where the younger medics, it's so refreshing, because they get it. Like the older medics, they weren't making that connect between, like, being a medic, and dealing with the medical issue; and dealing with all the other stuff… They would see somebody in their clinic: oh, the malaria films are out, okay, we better give them whatever they need to give them and send them on their way. And they weren't considering… Can they even afford to get that medication?... all the other stuff.’ *P13* | |
| *Civic-mindedness: giving back, pro-bono work and social justice* | | |
| Quote 12 | ‘part of my motivation for doing medicine… was this feeling of wanting to make a difference or contribute or... and of being fortunate. And therefore, I think that's part of my family background. You know, that you're aware of your good fortune or your privilege in being safe enough and comfortable and educated and all those things and supported. And to me, I guess I just grew up… with that sense.’ *P28* | |
| Quote 13 | ‘I really feel like we as doctors… are given a very privileged position in society. People listen to us, they respect our words because they trust that we are trying to help people and that we are fairly highly educated. And also like the government, you know, helps to give us this education and the universities have given us this education. I think we do have more of a responsibility to give back to society rather than just advance our own personal agendas. But I also understand that not all health professionals share that belief.’ *P21* | |
| Quote 14 | ‘So, I like to work, or I feel more comfortable working I suppose, with people who perhaps have the greatest needs and the least ability to influence their outcomes. And I kind of think as someone who's been fortunate enough to be given the opportunity to go to university and to earn a good income, that it is a responsibility both personal and professional, to give back in some way.’ *P31* | |
| *Advocacy: a personal and professional duty* | | |
| Quote 15 | ‘I think [advocacy is] part of a doctors' moral compass. That should be one of the guiding things that propels us forward.’ *P31* | |
| Quote 16 | ‘Advocacy is a massive part of this field.’ *P13* | |
| Quote 17 | ‘I think that is, that is, in part of our medical ethics, that we have a duty to advocate, that is in part of our medical ethics. Not everyone agrees with that. But certainly, I think we have a duty not only just to our individual patients, but to the public as a whole. And I would include people you know, detained by the government, in the definition of Public. But not all doctors agree with me on that, even though it is in our medical ethics standards. I think that whilst we might be employed by any particular private or government organization, like I said earlier we have a higher calling; that is to medicine, to our profession, and that includes responsibility to everyone. And that's why we can't, the same reason we can't just prescribe whatever antibiotics we want to anybody, to our individual patient. Even though it might, it might benefit that patient... but on an individual level - on a population level obviously its bad news and can cause antibiotic resistance. It’s the same kind of rationale. I mean we've got to look after everybody right? Not just individuals, and not just ourselves.’ *P10* | |
| Quote 18 | ‘Advocacy is part of a healthcare professional’s responsibility.’ *P03* | |
| Quote 19 | ‘In my view, it's pretty clear that the code of conduct for doctors means that you do whatever you can, using your sphere of influence, to protect the health of individuals and communities.’ *P30* | |
| Quote 20 | ‘For doctors, I think you have that duty that because people trust you and because you have a voice, you have that duty to use that to try to influence people's opinion in the right direction.’ *P25* | |
| Quote 21 | ‘I mean, we were criticized by the Minister for immigration at the time for, you know, he criticized doctors for being advocates. Well, my answer to that is, doctors are advocates. That's a key role of a doctor. So, you know, whether it's advocating for children in immigration detention, to have adequate health services, or advocating for my patients here, we do it all the time. So, we'll advocate for services or for prevention immunization programs, or for access to special drugs, or for new services or for more beds in the hospital, or, you know, that's all advocacy. And it's a, it's just an integral part of being a doctor, just like a lawyer would be an advocate for them. You know, it's a, it's, I think it's integral. And I think if you work in a field, like paediatrics, particularly that the social justice issues are very pertinent and very evident, because we're often dealing with young families and disadvantaged families. And, you know, when you're dealing with sick, sick children, you sort of feel both the personal desire and a professional desire to advocate for them.’ *P04* | |
| Quote 22 | ‘Well I think they can't be detached, really.’ *P04* | |
| Quote 23 | ‘It’s kind of intertwined I think. My personal ideals tend to include professional obligation.’ *P31* | |
| Quote 24 | ‘Who I am, as a psychiatrist and who I am as a person is the same thing there are obviously things that I do as a psychologist, that I don't do as a father or a citizen or whatever and vice versa. The ethical principles that underpin my psychiatric work are the same ethical principles that underpin my life outside psychiatry. If that doesn't sound too sanctimonious.’ *P23* | |
| Quote 25 | ‘It all goes together and it's the same. It goes on the same line of, you know, my personal beliefs, my code of ethics, and my professional, you know, boundaries and rules. There is no difference between that. It's very simple.’ *P11* | |
| Quote 26 | ‘It’s really hard to distinguish where the personal values and where the professional values intersect because the personal values are why most people choose to become doctors in the first place. And especially when we’re studying and being medical students from a very young age, like our medical education like irrevocably helps, like shapes our moral and ethical conscience along the way as well. And it becomes very hard to separate the two because I think, when we think of ourselves as doctors, it doesn’t just stop after working hours: it becomes a bit of an identity thing as well. And kind of it extends much further into our lives than that.’ *P21* | |
| Quote 27 | And I think it's hard to pick apart those two things. I think that, I think as you train and as you get older, those things become intertwined. You know, and, and, ultimately, I think it's your own experience of right and wrong, that you know, trumps everything. You, your knowledge of what's right and wrong as a human-being trumps what your professional opinion of what’s right and wrong, and I think that's the way it should be. Because I think… the professional elements can sometimes constrain you and your ethical response, which is why I think it's more important to concentrate on what you know, to be right and wrong as a human being.’ *P18* | |
| *Initial shock and outrage* | | |
| Quote 28 | ‘I just think I was appalled by the harmful effects of mandatory detention and not just on children, on the parents as well.’ *P03* | |
| Quote 29 | ‘…it was pretty shocking, the environment and... and the state that they were in. The despair… the pervasive distress of everybody.’ *P28* | |
| Quote 30 | ‘It was shocking experience. It was completely humiliating, I was stripped of my human identity I was marked with a permanent marker, was a number 982. So, it didn't matter whether I was a surgeon or a doctor because we were all treated like animals… the treatment was extremely harsh, was unnecessary…’ *P11* | |
| Quote 31 | ‘you can’t just make somebody better, who’s suffering from post-traumatic stress, if you’re going to send them back to the place that caused the post-traumatic stress in the first place.’ *P18* | |
| Quote 32 | ‘in my experience, we weren't given what we needed to do the job that we wanted to do. And we were sort of directly undermined at every turn.’ *P10* | |
| Quote 33 | ‘…there was no appropriate processing, there was no appropriate transfer process… transfers were being delayed deliberately, by, or it would appear to be a deliberate policy by the government, by making sure every single case, unless it was life threatening - immediately life threatening, which was the government's policy, was, was dragged through the courts.’ *P30* | |
| Quote 34 | ‘Everything from like, doubling up on medication charts and medication administration charts. The loss of information, the... the unwieldiness of it all, it was, it was just crazy. Like I can't... I've used some terrible, terrible medical records keeping systems but nothing like that.’ *P12* | |
| Quote 35 | ‘But it was that experience of being really challenged about my own professional opinion. So, I couldn’t say, ' You know, I'm a consultant paediatrician. This is what should happen.’ *P18* | |
| Quote 36 | ‘And the reality is, the client was not the patients, the client was the Department of Immigration and Border Protection.’ *P14* | |
| Quote 37 | ‘And so, I was shocked when IHMS made it quite clear to me that their duty was to the government. And that's what they worried about. And so, I said, well, in that case, you know, it's very clear to me that you're not acting in good faith here and you're not acting in the best interests of the patient. So, I'm not coming back. You know, I'm not going to be part of this then.’ *P14* | |
| *Mandatory reporting* | | |
| Quote 38 | ‘And so, whenever you’re a paediatrician, you cannot discharge a baby into circumstances that are unsafe. That's just the way it is. And what normally happens in that case, if you think that a baby is going back into circumstances that are unsafe, you would get family Community Services involved, and that would not happen. And it's very clear what your responsibilities are in that usual situation. But, you know, we all know what the rules are on child protection. But in this situation, it was as if the normal rules didn't apply. And the water around child protection was just completely muddied.’ *P18* | |
| Quote 39 | ‘So, in Australia, there's Child Protection frameworks. So, you would report it to them, we're mandatory reporters, it's your duty of care, to report a child that's telling you they've been abused, or a mother that's telling you they've been abused, and they've got one of your patients in their care. You are a mandatory reporter, and you have an obligation to do something about that. The problem on Nauru is there's no child protection framework, there's nowhere to report that abuse. So, you're sending your patients back into an environment where they're going to be abused again.’ *P13* | |
| Quote 40 | ‘And that experience of immigration, out ranking health…and I'm not used to that, I'm not used to, to people saying, when we're talking about a health situation, that there are other things that are more important than the health of the baby. And that experience, I suppose, changed my view on how much we needed to do to ensure that people got good health care, and that we needed to be more vocal about it and that I needed to be more involved.’ *P18* | |
| Quote 41 | ‘Mostly whistle-blowers have gone in there not intending to be whistle-blowers, having a belief of some kind of that it was okay, on some level that it was okay, or, you know, “I’m just doing the job. Someone else would do it. How bad can it be?” And, and getting so shocked that, like me, they come out and advocate.’ *P16* | |
| Quote 42 | ‘It started out as being all about individual kids and families that I was seeing, then it became much more of a political exercise.’ *P23* | |
| Quote 43 | ‘I just pushed it up the chain or I raised my concerns. And I was doing that too at first too. It was only when I realised pushing up the chain didn’t make a difference. I thought, hang on, well I’ve got to say something now.’ *P15* | |
| Quote 44 | ‘We decided to, we have no alternative but to talk to the media. What else were we going to do? Like, we've reported it to the directors, we can't go much higher. We couldn't go much higher than that. Apart from to Peter Dutton. And Peter Dutton is the one that's putting them there. So… where's the chain of command? You know, you report it to IHMS, IHMS have got a contract with the government. The government are the ones that are putting them there. So, it's just the cycle of, there's nowhere else is there?’ *P13* | |
| *Guilt, complicity and working within the system* | | |
| Quote 45 | ‘I felt really corrupted by the experience. I still do feel complicit in what was going on there… And no matter what... how much advocacy I do, that's always going to be the case… I felt a lot of guilt and I was ashamed.’ *P10* | |
| Quote 46 | ‘I felt such guilt, complicity by being there as a paid employee… Well, you’re supporting the machine, aren’t you? Even if you’re trying to do something right while you’re there, which is pretty hard, I’d say near impossible to do real work there… And all I could think of was, I’m complicit. I’m like the Nuremberg trials where they said they were just doing their job.’ *P16* | |
| Quote 47 | ‘If we're going to be responsible psychiatrists going into that environment. The only role you can have is as an advocate. because otherwise you're colluding with the system… I don't know whether I would have called myself an advocate. But that was the role that I saw as being the only ethically defensible position to take.’ *P23* | |
| Quote 48 | ‘I feel responsible for letting the system function and I think it’s a really evil system.’ *P19* | |
| Quote 49 | ‘[It’s] not tenable for medical professionals to be involved with this, it amounts to a form of complicity with their treatment, by continuing to allow our labour to be used in this fashion. And that... the end of the end of that realization is that then we must no longer be complicit. We can do that by ensuring that these people are not treated like this. And if we, we cannot do that, then we must no longer be involved.’ *P12* | |
| Quote 50 | ‘I don't see how you can do [work in immigration detention]. I mean, you can't provide completely substandard care, and not being able to treat people properly and not even treat them as human beings, you can't do that!’ *P24* | |
| Quote 51 | ‘I don’t think it’s defensible for health professionals to go and work for those contracted organisations.’ *P21* | |
| Quote 52 | ‘I don't know how any paediatrician could go and see what I saw and not think it’s a bad thing. I don't see how that's possible.’ *P14* | |
| *Becoming an advocate: proximity* | | |
| Quote 56 | ‘the invitation to join the commission to go to Christmas Island; that was opportunistic… So, all of those things are sort of opportunistic. And I guess, you know, as a doctor, we're very fortunate in having skills that are transferable, whether they're educational, or advocacy or clinical skills. And so, it's a matter of, because I'm in an academic job, I had the capacity to respond to those opportunities. But I could easily have not done it.’ *P04* | |
| Quote 57 | ‘I had a friend at the time who was working in South Australia. She’s a psychologist. And she said, “Come down! They need somebody.” I said, “Okay, great.” And I had very little knowledge about the centres or about what was going on.’ *P19* | |
| *Becoming an advocate: readiness* | |  |
| Quote 58 | ‘I'm in a position where I can take a strong gravitational stance. Partly because, you know, my career's not at risk. You know? If somebody sacked me tomorrow, that's not the end of the world for me. I'm kind of set up for retirement if I want to. I've got a kind of freedom to say things that you haven't.’ *P23* | |
| Quote 59 | ‘Some working health professionals are perhaps a bit more reluctant to advocate because of the fear, whether real or imagined - I do acknowledge it is real - of professional repercussions. So, people, for example, feeling like their workplace won’t support it, that even people like [redacted] being, in the past, being threatened by, under the Border Force Act for speaking out of fear of jail-time imprisonment. Really quite serious, like, ramifications. I think being in that working position means that they have to think more carefully about that and I think, as being young, we might not have those things over our head yet. So, we might feel more inclined to be free to speak, to speak out about our opinions as well.’ *P21* | |
| Quote 60 | ‘Doctors are worried that they're going to get pulled up, you know, insurance wise, they're worried about whether they're covered by medical insurance, indemnity. They're worried about whether they're stepping outside the realms of their profession.’ *P13* | |
| Quote 61 | ‘And also, I’m not an Australian citizen and so, erm, that was a worry. I’ve got a permanent residency but that can get revoked if Peter Dutton decides he doesn’t like me. So that’s a bit of a worry.’ *P15* | |
| Quote 62 | ‘There was a colleague, who doesn't yet have permanent residency. So, although that person was in a very good position, you know, does a lot of other medico legal reports, very interested. That person…. offered to help me, but they weren't going to put their name on it.’ *P28* | |
| Quote 63 | ‘were you ever scared? Of all the impacts this might have?’ *Interviewer*  ‘Yeah. Yeah. Heaps. So, look, I've got a daughter. She's tiny, she's at the time like 5 or 6, so like, going to jail is not something you want to do if you've got a kid.’ *P12*  ‘Yeah.’ *Interviewer*  ‘And I've got responsibilities, like, you know, mortgage and all the other normal things other people do and jail is really inconvenient for all that stuff. And I really thought that, like, it's funny now, because now I look back, and I go, that was never gonna happen.’ *P12* | |
| *Personal satisfaction* | | |
| Quote 64 | ‘I think I realised that I had a need within myself to do something like this. So, you know, I sort of… got further involved. And I've got to say, I really do enjoy the work now. So, no I don't think I ever really saw myself doing all this. But now that I'm involved in it, I'm enjoying that involvement. And particularly, as it's such a contrast to the practice where I work in where I see a lot of very advantaged people. You know, I see a lot of man flu, for example; the worried well. It's sort of almost a relief on Monday afternoons when I turn up at [redacted: a refugee clinic] and I see people who really do need my help, [rather] than people who only half need my help. I do get a lot of personal satisfaction in doing that, and that's what, sort of, life's all about.’ *P25* | |
| Quote 65 | ‘There's a little bit of kind of gratification out of it, isn't there? I mean, you don't do this work, unless it makes you feel good. Let's be honest about it. I mean, everybody can say, Oh, I'm just, you know, I'm only doing it for the patient. Like you are, but you do it because you want to do it. And because it makes you feel nice as a person.’ *P13* | |
| Quote 66 | ‘What was it that made you keep going with the pro-bono work?’ *Interviewer*  ‘Oh, because it’s worthwhile, why else?’ *P09* | |
| Quote 67 | ‘I've been driven really by, just by the interest and the opportunity and the challenge of these things. And all of them, have been a huge amount of extra work on top of my work, and family and everything else, but, but they've been really the most rewarding parts of my career, because, you know, they just give you entree into worlds that we would not, you know, have, have entry into otherwise.’ *P04* | |
| Quote 68 | ‘I'm completely sold on the area [of advocacy], because it's such a fascinating and important area to be part of.’ *P20* | |
| *Self-preservation* | | |
| Quote 69 | ‘But it would have been harder for me to live with myself if I hadn't done anything, than if I had. So, it was actually in my interests in a way, to say something, because I think the guilt of not saying something would have cost me more sleepless nights than the stress involved with saying something. So, in a way, it was self-preservation, in a way.’ *P18* | |
| Quote 70 | ‘Why you're motivated to act? Partly, I think it's self-preservation.’ *P28* | |
| Quote 71 | ‘What I’ve heard from many doctors is that they've been feeling powerless. And it didn't sit well with their ethics. And… many of the doctors who are doing these cases are so appalled by the condition of some of these patients that they are becoming quite distressed by it, but at least they feel that they're able to do something to address the situation by using this legislation.’ *P30* | |
| Quote 72 | It, it was almost like, not that I’m so noble or anything but, if you were faced with a life-threatening situation and you had to break out of your house, and smash the windows or something, you wouldn’t think, “Oh God, I’m wrecking my windows,” would you?’ *P16*  ‘No.’ *Interviewer*  ‘It was sort of like that… I felt, I felt, in a way, that I was, my integrity was so under threat I was gonna be on the wrong side. And, if there’s one thing I learnt from my parents’ holocaust history it was, you know, Nuremberg will happen one day. And, and, and even that part was like, “I’m gonna be one of the perpetrators. I’m gonna be the one who has to justify why I did it, why I was part of it.” That’s how I saw it. I felt much more vulnerable. I felt I had to dissociate. So, it’s not as noble as it, I’d like it to be, but I had to disassociate from it.’ *P16*  ‘Yes, yeah.’ *Interviewer*  ‘And, of course, I felt for the refugees but the, the most, the strongest feeling was of contamination with, with an evil process. That was … I just couldn’t do it.’ *P16* | |
| *Personal ethics* | | |
| Quote 73 | ‘I think it’s my personality and it’s a sense of justice.’ *P16* | |
| Quote 74 | ‘It's been a sort of an issue that… hasn't gone away for me… it's one of those things that I think just remains with me and it's like a burning kind of concern, basically, that kind-of keeps eating me up.’ *P08* | |
| Quote 75 | ‘I knew that I should speak out… it’s in my bones.’ *P07* | |
| Quote 76 | ‘I think there is a moral obligation for healthcare staff to speak out when they are confronted with something that is just unacceptable.’ *P15* | |
| Quote 77 | ‘And I think we have a duty as citizens and as doctors to be involved in those things that are a problem for Australia, whether it's in climate change, or what's going on with refugees or any other matters. So, I think it's, I think it's wrong to be quiet, when you can see something happening that's wrong. I think, my belief is that you need to speak out and do what you can to try help and correct that situation.’ *P25* | |
| Quote 78 | ‘For us to not say or try to do anything about it would be, almost be a form of neglecting our duty of care to help those people.’ *P21* | |
| Quote 79 | ‘I think, I mean you’ve signed up to help and heal people in a way, most fundamentally. I do think, you know, first and foremost, you have this obligation to your patients, but you also do have an obligation I think to, to speak out against these, these sort of things as well.’ *P19* | |
| Quote 80 | ‘I think [whistleblowing] needs to be seen as a more important thing to do and not doing it as soon as something is negligence.’ *P01* | |
| Quote 81 | ‘I think if those repercussions weren't so serious I think doctors would have, every doctor who's gone, would have a responsibility to speak out. It's just difficult because of the consequences you'll face.’ *P29* | |
| *Alliance building* | | |
| Quote 82 | ‘The most powerful point was when there was collaboration.’ *P28* | |
| Quote 83 | ‘We’ve joined forces because the legal profession is very powerful… It’s excellent. It’s an excellent combination.’ *P16* | |
| Quote 84 | ‘Probably one of the most positive things that’s happened [is the collaboration between doctors and lawyers advocating for asylum-seekers.] That was a major concerted partnership. And I think they've been great, great partnerships… without them working collaboratively, we wouldn't have got the results that we have.’ *P05* | |
| Quote 85 | ‘We have common cause with other disciplines in trying to think about a viable solution for this for our country… we have to, we have to get some joined up thinking happening…we're going to have to collaborate… I think we, we have to get smarter at working together to, to have an effect.’ *P08* | |
| Quote 86 | ‘I think there needs to be a body of a voice, a collective voice, for it to work. I think individual, you know, strident individuals, harping on about the same thing, get pigeonholed. And I think that's a real problem in refugee health. I think there needs to be a number of voices, different voices, it can't just always be the same voices, because if it is, they lose power. Whereas if you get enough different voices and different health professionals talking, then it is powerful. But not just not just the refugee advocates, it can't just be them, it’s got to be other specialties, as well.’ *P18* | |
| Quote 87 | ‘It can't be just doctors, doctors alone to not bring about a social movement. It’s very important that we add our voice to things. But what's even more powerful… it's about the partnerships I think. Social movements are not one group, they have to be everyone. If you look at what's going on in the states, or Hong Kong, which is a fundamental human rights group, its mass movements across different groups. And the student voice is very important in that. So, I think, I think it's how we collectively connect together at the moment, and otherwise the government just divides and conquers.’ *P05* | |
| *Collegial backing and support* | | |
| Quote 88 | ‘And the various colleges have come to the party, you know, at different times. And that lends a lot of credibility, because when you have the peak doctors body, and when you have the people that the colleges who are basically the gold standard of what's acceptable clinical practice, all saying the same thing, then it's very hard for the doctors to be called activists if they are going against what the government says, because they're going against the undisputed, at least on paper, the undisputed advice from the specialist in their field. It's not just something that they've dreamed up because they are a troublemaking activist.’ *P02* | |
| Quote 89 | ‘The AMA, in particular has, you know, from time to time been a very, very potent voice when it chooses to step up… And I think it's really important that the medical profession does have a say and shows leadership in these important social policy areas.’ *P30* | |
| Quote 90 | ‘But I do think overall, that… the Australian Medical Association, those bigger entities, and these organisations do have a responsibility to take a stance.’ *P29* | |
| Quote 91 | ‘For the AMA; refugee health is not their top priority. And it does, it needs more leadership I think. But they’re also complicated by the fact that not everyone agrees that this should be the top, the most pressing issue. It’s a bit of a catch–22. But I think sometimes you need strong leadership.’ *P21* | |
| Quote 92 | ‘I mean, and so look, the AMA, and other organizations, so the college's for example, they’d all stopped short of actually doing something. They were more than happy to put out yet another position statement, saying, “This is not good for the mental health of children and pregnant women shouldn't be blah blah blah.” Like, it's just like, it got me just frustrated as hell.’ *P12* | |
| Quote 93 | ‘I feel like the AMA recently went backwards… I think there was a point when, yeah, they just stepped back, got scared or thought about their funding or became more conservative.’ *P28* | |
| Quote 94 | ‘So, I think, I think the problem with the medical community has always been that they didn't necessarily think that there was a voice to be heard. I think for a long time, a lot of big players, and I'm going to point the finger straight at the AMA, have really dominated the scene in terms of being the voice for doctors, to the point where individual doctors didn't actually feel like they could make their opinions heard. And so, I think it wasn't, you know, the AMA still hasn't come out and supported any of this sort of stuff, because they are a lumbering bureaucratic organization, who are perpetually trying to work out where the political coin is best spent. And the question, they're a doctor’s union. And I think if we continue to think of them as anything other than a union, then we will be silly, because their priority is not the health care of this country, their priorities is the welfare of doctors in this country… I think when it comes to broader health issues, we potentially need a completely different system. Maybe we need a you know, the American equivalent of the surgeon general, who oversees health policy. An expert, you know, not the health minister, but an actual doctor, who is responsible for health policy or who serves as an advisor, because it's a role that doesn't exist. and I think the medical community needs to feel empowered to stand up about the sorts of issues.’ *P06* | |
| Quote 95 | ‘Doctors are, are multifaceted people, you know. There’s … there’s no model for what a doctor is or believes.’ *P17* | |
| Quote 96 | ‘And that's part of the problem with the whole, I think, response from the medical fraternity. From the medical fraternity's point of view there is this huge range of attitudes. I don't think we'd ever get to [a consensus about that] I'd love us to, but I don't think we would.’ *P10* | |
| Quote 97 | ‘The importance of big important organizations behind you rather than being a lone voice, which I increasingly believe is very important for your own safety and also for the strength and helpfulness of the message.’ *P26* | |
| Quote 98 | I want to sit within the profession, I don't see myself as going out on a limb. I won’t do that. I just think the solidarity is really important. I think we have a common tradition, I will honour my colleagues at all times.’ *P08* | |
| Quote 99 | ‘I think that we shouldn't underestimate the impact of some of this work on individuals. I think that, you know, advocacy and, and working in difficult situations can be quite lonely. And I think that's why we're lonely, if people disagree with what we're saying, for the first thing, lonely, if you feel that you're a lone voice lonely, if you get a lot of criticism from the, through the media or from government. Lonely if your hospital and your institution doesn't support you. So, you know, I think that, that's where these groups of like-minded people, like, Doctors for Refugees, are really helpful.’ *P04* | |
| Quote 100 | ‘I think that hurt the most... was the criticism that we all experienced from our own people.’ *P13* | |
| Quote 101 | ‘It’s so hurtful… [After engaging in advocacy actions] I then got a disciplinary letter after that. They said that, that I had gone out and... used my position to protest… So yeah, so they essentially were trying to fire me… It's been really hard.’ *P13* | |
| Quote 102 | ‘That's one of the things that the executive have criticised me and us for, they've said, Oh, you're too political.’ *P13* | |
| Quote 103 | ‘But for me that first experience was absolutely terrifying. I didn't sleep for weeks, like I was so stressed. Because it wasn't just the, it wasn't just the department. But it was also, you know, the hospitals response initially, was very much 'Oh, don't rock the boat, you know, don't make trouble'… And it was really difficult. And eventually, the hospital did kind of come on board, and supported me. But initially, it was very much I felt like I was on my own at the beginning. And I just felt as if it was me, and people thought that I was making a fuss. And I was basically saying, look, all I'm doing is my job. And, you know, people shouldn't be put under this amount of stress for effectively just doing what they're employed to do, which is to look after children. And to ensure that children are, you know, treated appropriately and protected. And eventually, they did come around the hospital did come around, but I find that very stressful as well, just having to negotiate with senior hospital administration and negotiate with Australian border force.’ *P18* | |
| Quote 104 | ‘But some people get very frightened... I had colleagues say to me you'll lose funding for hospitals, if you carry on like this because government won’t like it.’ *P05* | |
| Quote 105 | ‘Like this is what [the hospital executive] said: we really admire the work you're doing. But don't ever mention the hospital when you're speaking publicly…. “So, why are you saying that we shouldn't mention the hospital in our advocacy?” “Oh, because you know, the Liberal government fund our hospital. And this could affect the funding of all the other services. And this puts us in a very, very difficult position, if you're going up against the government.” And then I've got to go and have meetings with them.’ *P13* | |
| Quote 106 | ‘So, what's the secret to successful advocacy? I don't know. The secret to the much more personal, selfish thing, if you like, of sort of surviving or flourishing as an advocate. I think you’ve got to find yourself a peer group.’ *P23* | |
| Quote 107 | ‘If I get upset about something, I've got a couple of really good colleagues who are doing the same work, who I can ring up straight away.’ *P09* | |
| Quote 108 | ‘I developed the Australian Paediatric Refugee Health Network… and we can meet face to face once every six months. And talk about cases. We can talk about advocacy, we can talk about all the stuff that's happening nationally. And it's been really good, really helpful and really supportive.’ *P13* | |
| Quote 109 | ‘There’s a culture of advocacy amongst medical students… [there’s] strength and safety in numbers, and, when you find other people who think similarly to you, it really empowers you to feel like you can speak up and advocate and engage.’ *P17* | |
| *Defining medical advocacy and its scope in systems of abuse* | |  |
| Quote 110 | ‘But I think we would have to be very focused on medical stuff… I feel that when doctors say, make the statement, and it's purely medical, we get - we have to - we get respect that way. Then when we start straying into UN regulations, and what goes against maritime law and habeas corpus and stuff that, you know, we may know reasonably well, but we're certainly no authority on it, then we lose credibility, because then it just looks like we're just parroting some ideology, rather than talking about our area... talking within our area of expertise.’ *P02* | |
| Quote 111 | ‘I've assumed that my advocacy… I've assumed that the expert position that I speak from is more powerful when it's based on my professional expertise. And therefore, I think it hasn't been separate from my road as a child psychiatrist.’ *P28* | |
| Quote 112 | ‘So, all of the work with Kids Off Nauru with like recruiting or getting doctors involved in advocacy I think was successful because they said, “We are advocating for the healthcare outcomes of children who are on Nauru.” It wasn’t a political platform. It had nothing to do with human-rights violations in terms of indefinite detention… It was exclusively about the healthcare outcomes of children who were being held in offshore detention without access to healthcare and with like palpable… poor health outcomes directly as a result of their detention. And I think the fact that it remained singularly healthcare-focused meant that the message was more effective because people listen to doctors about healthcare. People probably listen to doctors about non-healthcare-related things too, but they will certainly listen to you if you start talking about healthcare. And doctors were more likely to get involved ‘cause they didn’t feel like they were engaging in a political campaign: they felt like they were engaging in a, in a health-advocacy program, not a political movement. And I think that was key…. That’s what made it kind of palatable for the public and the politicians. And it’s what made it kind of more accessible for doctors.’ *P06* | |
| Quote 113 | ‘In general, I guess a lot of the media and other advocacy has been really, I've tried to stick to my role as a paediatrician, health professional. In other words, to advocate for access to appropriate health care and preventative care for children in immigration detention… Because I think that if you cross the line and start becoming, you know, I don't have the answer to the immigration problem, to the refugee problem.’ *P04* | |
| Quote 114 | ‘Well my view very much is the medical advocacy role is very much at a professional level. So, I see advocacy as a core element of what it is to be a doctor… And that's different to being someone who would be shouting at a rally or chaining themselves to a tree or you know, something like that. I don't see that as being our role, I see our role as highlighting what the medical issues are. So that people can make informed decisions… And it's the same with the science of climate change, or whatever, I think, it’s very important that scientists are just reporting scientific facts. And not, you know, over egging it, if you like. Because as soon as you become an advocate in, you know, an organisation like Getup! or something else, I think you lose the power of being a professional advocate. Because a professional has some standing and some expertise only in their area. And that is where I think professional advocates should be, that space that is occupied by their professional expertise.’ *P14* | |
| Quote 115 | ‘I think more nuanced ethical guidelines [are required] I think they need to acknowledge the shortcomings and trade-offs of working in there ‘cause, you know, every guideline comes back to that thing: put your patient first. How? You know, how? How are you supposed to do that?’ *P19* | |
| Quote 116 | ‘None of the professional frameworks that we operate under actually anticipate a situation where you are going to be advocating against a government policy. You know, they're mainly... it's more like you anticipate advocating based on evidence for something, or for more funding for something, you know. Like, they don't, our frameworks don't guide us, when its actually government policy that's causing the harm.’ *P28* | |
| Quote 117 | ‘That's the thing: it's what is advocacy? Right? And what are the boundaries around advocacy? So, for example, they might say, Oh, yeah, we expect you to advocate for your patients. That's expected. So, if there's a patient on orthopaedic ward that needs a wheelchair, we expect you to advocate to get that patient a wheelchair. It's how far you they expect you to go with advocacy. And so, their line is when you're lobbying government, and that government has an influence on the hospital. That's where they're drawing that line.’ *P13* | |

1. *The Invitation: to Tom Hughes* by Charles Kingsley (1819-1875). [↑](#footnote-ref-1)
